# There is No Consensus on Biological Sex

**DOI:** 10.1111/ele.70350

**Published:** 2026-03-05

**Authors:** Madeline G. Eppley, Andy Lee, Robert Dellinger, Ally Swank

**Affiliations:** ^1^ Department of Marine and Environmental Sciences, Northeastern University Marine Science Center Nahant Massachusetts USA; ^2^ Department of Biological Sciences Purdue University West Lafayette Indiana USA; ^3^ Department of Oceanic & Atmospheric Sciences University of California, Los Angeles Los Angeles California USA; ^4^ Department of Biology Boston University Boston Massachusetts USA

**Keywords:** biological diversity, gametic sex, multivariate sex, policy, science and society, sex and gender

## Abstract

There is ongoing scientific and societal discourse on the definition of biological sex. At this critical moment when misinformation about sex is being applied to policy globally, scientific clarification is valuable. Here, we evaluate the primary approaches to defining sex and synthesise the active discourse to conclude that there is no current consensus on a definition of sex that is free of assumptions and limitations. While there is no current consensus, we do not advocate for a single definition and contend that a lack of unanimity is not inherently problematic. No matter what definitional choices are used, we provide actionable recommendations to improve accuracy when describing sex. Most importantly, regardless of scientific debates, no biological definition of sex should be used to dictate human rights.

## Graphical abstract

At this critical moment when misinformation about sex is being applied to policy globally, scientific clarification on the definition of biological sex is valuable. Here, we evaluate the primary approaches to defining sex and synthesise the active discourse to conclude that there is no current consensus on a definition of sex that is free of assumptions and limitations. Most importantly, regardless of scientific debates, no biological definition of sex should be used to dictate human rights.

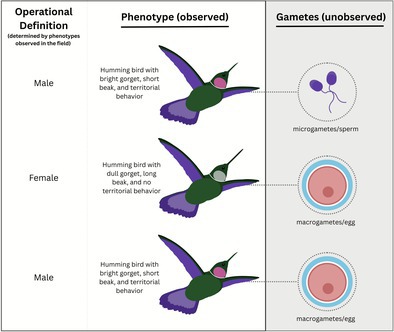

## 1 Main Text

Philosophers and biologists alike have debated for decades over the definition of biological sex (Griffiths 2020; Griffiths and Spencer 2025; Lewis and Sharpe 2023; McLaughlin et al. 2023; Roughgarden 2004; Subramaniam and Bartlett 2023), not unlike debates on what defines a species (De Queiroz 2007). As biologically inaccurate sex definitions are being applied in policy contexts around the world (e.g., recent U.S. executive order 14168, which aims to categorise sex as a binary and immutable fact of biology), critical scientific evaluation is particularly timely (Sudai et al. 2022). Evolutionary biologists and ecologists are uniquely suited to evaluate definitions of biological sex as scientists who investigate causes and consequences of sex and document the diversity of reproductive systems in nature. Current discourse among scientists aims to identify a single definition of sex. Our perspective is that no single definition of sex is free of limitations and assumptions, nor can any be universally applied without exception. Here, we focus our discussion on animal sex and do not address the diverse reproductive strategies found in other domains. We believe that acknowledging limitations and assumptions of current definitions of sex will lead to more accurate science.

## 2 Current Approaches to Defining Sex

Gametic sex: Gamete size has long been argued to be the most parsimonious definition of biological sex (Bateman 1948; Griffiths 2020; Roughgarden 2004). Under the gametic definition, sex is defined solely on gamete size (i.e., anisogamy), without considering other traits. Small gametes (microgamete) are defined as sperm and considered ‘male’, while large gametes (macrogamete) are defined as eggs and considered ‘female’.

Multivariate sex: The multivariate framework to define sex considers multiple traits used across various fields (DiMarco et al. 2022; Massa et al. 2023; McLaughlin et al. 2023; Miyagi et al. 2021). These traits can be primary sex characters (e.g., gonads, reproductive organs) or phenotypes that often (though not always) differ between the sexes (e.g., morphology or behaviour), and may have considerable within‐sex variation.

Sex eliminativism: Another school of thought is to eliminate the concept of sex altogether (Watkins and DiMarco 2025), but this approach constrains understanding of biological patterns and diversity (Aanen et al. 2016; Chen et al. 2025; Griffiths and Spencer 2025; Pape et al. 2024; Savolainen et al. 2024; Velocci 2024). While all approaches to defining sex have limitations and make assumptions, we contend that rigorous research on sex‐based variation remains vital. It also challenges simplistic and harmful ideologies of the sex binary (Pape et al. 2024; Richardson 2022). Thus, in this manuscript, we focus our discussion on the two primary debated approaches to defining sex: gametic and multivariate.

## 3 Discourse Within Ecology and Evolutionary Biology

Advocates for defining sex using the gametic binary argue nearly all animals produce two binary (micro and macro) gamete types; it is conceptually simple. Under this framework, sex‐related phenotypes (e.g., genetic, morphological, or behavioural traits) are not delimiters, but expressions of biological sex (Roughgarden 2004). Thus, an organism of a particular gamete size can exist in any region of phenotypic space because gamete size is unrelated to downstream phenotype (Griffiths 2020; Griffiths and Spencer 2025).

Advocates for defining sex with the multivariate framework constructed it to “avoid collapse of true biological variation into a single trait” (McLaughlin et al. 2023) and gain a multidimensional perspective of sex (Miyagi et al. 2021). Substantial interest in this framework has resulted in multiple recent publications and conference symposia (Data S1).

Recently, these definitional choices have come under intense scrutiny in academia and beyond. For example, the presidents of the Society for the Study of Evolution, American Society of Naturalists, and Society of Systematic Biologists (tri‐societies) published a public letter in response to U.S. executive order 14168 to the President and the Congress of the United States that advocated for a multivariate definition, which was subsequently retracted due to debates among tri‐society members (Boggs et al. 2025) (Data S1). A counter‐signature campaign by dissenting members advocated for a gametic definition (Coyne and Maroja 2025; Maroja 2025).

## 4 Why is There No Consensus?

**FIGURE 1:**
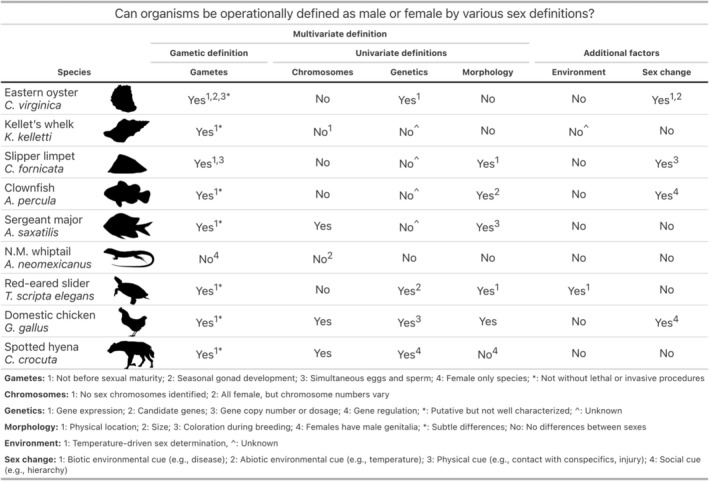
Can organisms be operationally defined as male or female by various sex definitions? The first column (“gametic definition”) indicates whether individuals can be classified as male or female based on observed gamete size (small = sperm, large = egg). The following columns (“univariate definitions”) indicate whether a non‐gamete univariate trait is a feasible operational delimiter of sex in that species: Sex chromosomes (identifiable, stable chromosomal differences correlated with sex), genetics (specific gene(s) known to determine sex) and morphology (observable traits reliably associated with sex). Environmental sex determination refers to sex being determined by environmental cues (e.g., temperature) while sex change cues indicate whether individuals can change sex in response to stimuli. While this table highlights common factors used to determine sex of individuals, it is not exhaustive in representing all sex‐based characteristics. We focus on animal species while recognising that organisms across the tree of life (e.g., plants, fungi) also share diverse sex characteristics. Furthermore, this table is reflective of current available knowledge and is subject to change with further research on these species. *, indicates limitations or exceptions explained in the footnotes; ^, indicates trait status is not yet determined or remains poorly characterised; no, the trait is absent or cannot be used; yes, the trait is present and can be used to operationally define sex.

While the gametic definition is simple in theory, it is difficult to use in practice, as many scientists do not (and cannot) directly observe gamete size in their system due to invasive techniques that often confer mortality. Scientists may understand biological sex using the gametic definition, but operationally use proxies of gametes to classify the sex of individuals. However, the operational use of proxies necessitates assumption (i.e., assuming a column in Figure 1 is a perfect predictor of gamete size). Additionally, this narrow definition is not inclusive of reproductive approaches beyond anisogamy (e.g., isogamy) and does not classify organisms before sexual maturity or after reproductive cessation as having a sex (Griffiths 2020; Roughgarden 2004).

Although many are proponents of a multivariate approach, in practice, scientists are still operationally defining biological sex using a univariate definition (e.g., using only one column in Figure 1). Additionally, different univariate traits may be chosen to define sex across studies within the same system, which may lead to a lack of reproducibility. For instance, to determine the sex of an organism, an evolutionary scientist might use gametes, a population geneticist might use chromosomes, while a field biologist might use behaviour. Exemplifying this potential mismatch, studies show a 3%–25% error rate when comparing various univariate sex definitions (e.g., gonadal, genetic, gametic and phenotypic) across salmonid species (Brown et al. 2020; Cavileer et al. 2015; Robertsen et al. 2025; Robertson et al. 2024). It is also worth noting that while one definition or trait may be operationally useful to determine sex in one species, it might not work for others. Some species do not have sex chromosomes and others only have one sex (Figure 1).

**FIGURE 2:**
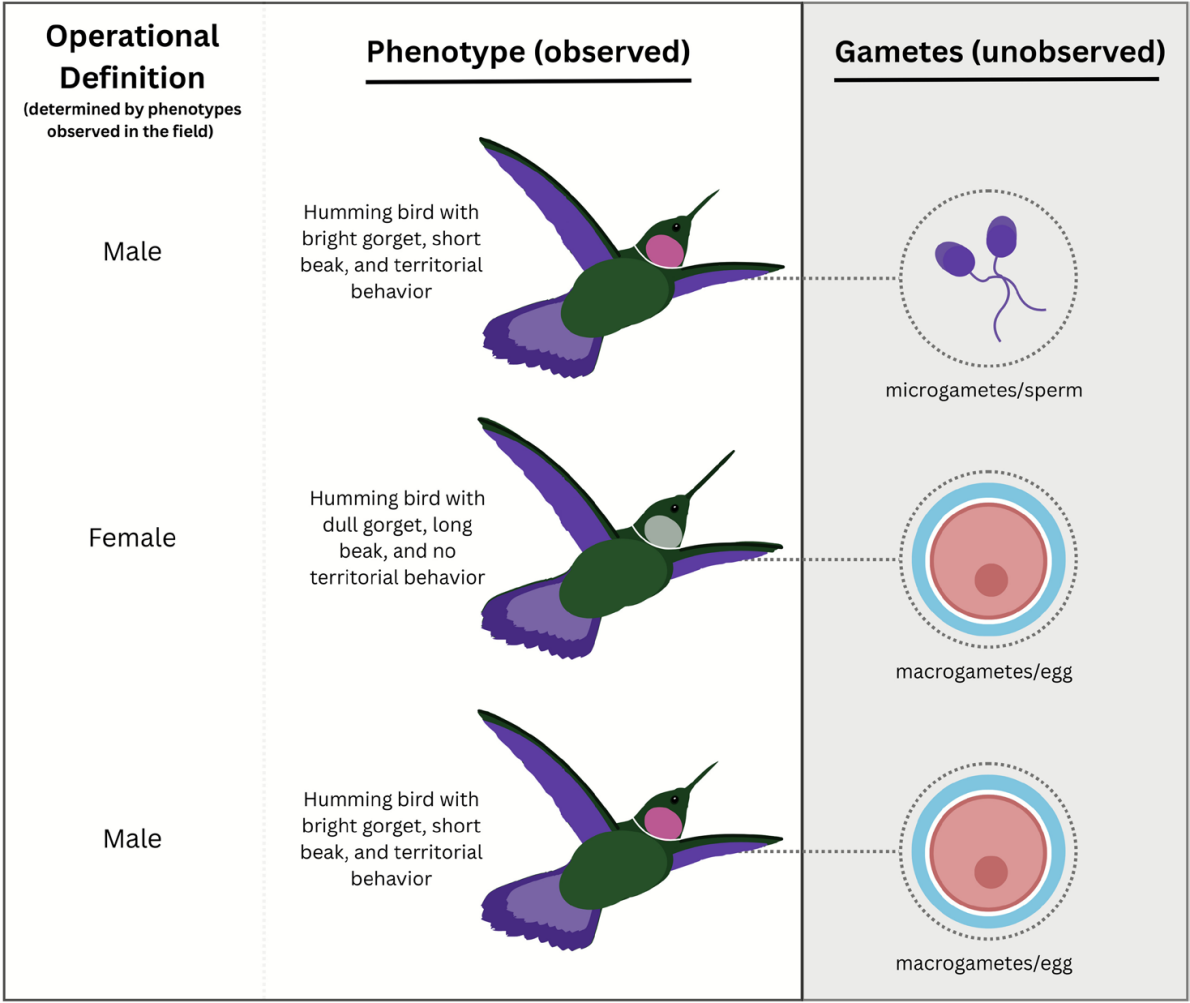
Operational biological sex definitions applied to hummingbirds (Bleiweiss 1992; Bleiweiss 1999; Bleiweiss 2001; Diamant et al. 2021; Falk et al. 2022; Falk et al. 2021) where sex is operationally defined by binary plumage phenotypes in the field. Each row depicts a wild individual hummingbird with morphological traits (beak length, plumage coloration), behavioural traits (territoriality) and their unobserved gamete type. This example illustrates how the same individual can be operationally defined as one sex in the field (based on external phenotypes) but classified as a different sex based on their gametes (which cannot be easily observed in the field), highlighting how definitional choices shape scientific reporting of sex.

Under the gametic or other univariate and multivariate definitions (Figure 1), scientists may still assume essentialism—the concept that all males or females share the same ‘essence’ which determines their behaviours, characteristics, or roles—leading to inaccurate classifications and interpretation of data (Carlen et al. 2025; Pérez 2025; Stern et al. 2018). Essentialism can cause scientists to wrongly assume predictive power between gamete size and other traits. For instance, individuals may display phenotypes of one sex while possessing gametes of the other sex, challenging sex essentialism assumptions (Figure 2). Different univariate definitions can thus result in the same individual being classified as a different sex. A hummingbird with shorter beak and bright gorget may be classified as male using phenotypic definitions in the field, but classified as female if its gametes could be examined in the lab (Figure 2, Row 3). Thus, the gametic definition should be applied when gamete sizes are directly measured (e.g., Gantt et al. 2025) because using proxies of gametes necessitates assumption.

Some traits may be very good predictors of gametic sex. In Atlantic salmon ( *Salmo salar* ), genetics can correctly predict gametes in 96%–97% of individuals (Brown et al. 2020; Robertsen et al. 2025; Robertson et al. 2024). However, even when proxies are strong predictors of gametic sex, scientists make an assumption about the sex of individuals when gametes are not directly measured. While only a small percentage of individuals represent mismatches in these systems, they represent important biological variation that should not be dismissed. Misclassification of sex can have implications for conservation and management (e.g., Hiller et al. 2014; Lesturgie et al. 2025) and obscure rather than illuminate patterns of interest (Figure 2).

Under the gametic definition, an individual that looks like a male and behaves like a male while possessing large gametes may have historically been considered “transgender” by Roughgarden (Roughgarden 2004) or “transexual” by Bleiweiss (Bleiweiss 1992; Bleiweiss 1999; Bleiweiss 2001): its sex is defined by its large gamete (female) while possessing traits associated with males of its species (e.g., Figure 2). Scientists can then describe this individual as female and ask: why does this individual look and behave differently from other females? However, we do not advocate for the use of terms like “transgender” or “transexual” in these contexts because they refer to the experiences of an organism (their gender identity) which cannot be understood. Recent research describes this phenomenon as male‐like ornamentation and female polymorphism (Diamant et al. 2021; Falk et al. 2022; Falk et al. 2021).

In the multivariate model, such individuals may be considered “intersex” (possessing various sex characteristics that fall outside the male–female binary) and may be described as a “bright‐plumaged egg‐producer” (sensu McLaughlin et al. 2023). Here, scientists can ask: why does this specific phenotype occur? What role does this individual play in the species' social system? While there is no current consensus on terminology for such individuals, it is nevertheless helpful to describe sex in some way because explicit description of sex allows scientists to investigate questions about individuals' roles in wild populations.

## 5 Action: Be Clear and Specific When Describing Sex

The fact that there is no current consensus of biological sex is not antithetical to science; in fact it is congruent with the scientific process. The scientific method involves discourse, discussion and re‐evaluation. However, the field urgently needs an ethical and reproducible approach for discussing sex in a way that upholds scientific rigour. To do so, we must recognise the implications of the language used, because word choices have real impacts on people and communities (Cheng et al. 2023; Eppley et al. 2026; Rice et al. 2025; Sudai et al. 2022).

The field can disrupt misinformation by developing clear standards for the ways in which sex is described. Academic societies can be on the forefront of these changes by leveraging their credibility to clarify the field's stance on topics such as the diversity of biological sex and establishing clear guidelines for discussion of sex in their journals and conferences. For instance, an editorial policy for articles where sex is categorically defined may require authors to state in their methods which variables were used to define sex in their study; reviewers and editors could then verify compliance.

We contend gametes should be directly observed when using the gametic definition. If proxies for gametes are used, their limitations and assumptions must be explicitly stated. Additionally, we advocate for descriptive and specific language that acknowledges the variation of biological sex. The following examples outline our recommendations as they would be written in a scientific manuscript:

1. Claim use of the gametic definition when gametes are directly measured.
  “We determine sex in our study using the gametic sex definition by direct macro‐ or microgamete visualisation in lethally sampled individuals.”
2. State the assumptions and limitations if proxies are used to represent gametic sex.
  “We use the *sdY* gene as a proxy for gametic sex, which predicts gamete size with 97%–98% accuracy. We acknowledge that 2%–3% of individuals may be misclassified; they represent important biological variation, but are not focal to the current research.”
3. Specify what categories *were* and *were not* used to define sex.
  “We used a hormone threshold to classify sex, with individuals below the threshold considered female and all other individuals considered male. While we collected data on mating behaviour and morphology of all individuals, those variables were not used to define sex.”
4. Acknowledge sex variation and the limitations of categorical choices.
  “We determined sex by feather coloration, where individuals with green crown feathers were considered male and individuals with yellow crown feathers were considered female. However, we also observed individuals that displayed mixed yellow‐green plumage feathers. These mixed‐phenotype individuals were excluded from statistical analyses, but we provide complete phenotype data in the supplement.”
5. Explicitly state why chosen sex trait(s) were the delimiter.
  “We observed gonads and identified mature testes, mature ovaries, or transitional tissues consistent with sequential hermaphroditism. We sorted individuals into three categories: reproductively functional females (mature ovaries), transitional individuals and reproductively functional males (mature testes). This classification allowed us to analyse morphological traits as a function of the individual's reproductive state.”
6. Avoid conflating sex with gender.
  “We used a multivariate framework and observed gametes (sperm, eggs, both, neither), sex chromosomes (XX, XY) and behaviour (territory‐defending, nest‐building). We avoid describing behavioural traits as ‘masculine’ or ‘feminine’, as individuals with any gamete and any chromosomal state may display either behaviour.”

## 6 Conclusion

Lack of unanimity on how to define sex is not inherently problematic and we contend that a universal definition is not necessary to pursue. Scientists should be specific and clear when discussing sex and sex‐related traits to advance more accurate science and discussions. Active debate and discourse lead to better understandings of complex biological diversity. Regardless of how sex is defined, research that documents and describes the diversity and variation of sex in nature should and will continue in biology (Aanen et al. 2016). Our ultimate goal is to prompt critical thinking and discussion about how we operationalise sex in research, encourage consideration of the ethical and political implications of defining sex and urge acknowledgment that there is no current consensus on a single definition of sex. Most importantly, we assert that biology should not dictate human rights.

## Author Contributions

Conceptualisation: Madeline G. Eppley, Andy Lee and Ally Swank. Visualisation: All authors. Robert Dellinger, Madeline G. Eppley, Andy Lee and Ally Swank. Writing – original draft preparation: Madeline G. Eppley, Andy Lee and Ally Swank. Writing – review and editing: All authors. Final approval: All authors.

## Supporting information


**Data S1:** ele70350‐sup‐0001‐Supinfo.docx.

## Data Availability

The authors have nothing to report.
