# Supplementary material for: There is No Consensus on Biological Sex
Source: Ecol Lett. 2026 Mar 5;29(3):e70350. doi: 10.1111/ele.70350 (PMC12962796; doi:10.1111/ele.70350)
Supplement: Supplementary file 1 — Data S1: ele70350‐sup‐0001‐Supinfo.docx. [file ELE-29-0-s001.docx]

**Supplemental Text File 1**

*Additional details regarding the discourse on biological sex within ecology and evolutionary biology*

**Conference Symposia and Related Publications:** Substantial interest in the multivariate sex framework has resulted in multiple recent publications, special issues, and academic conference symposia. The Society for Integrative and Comparative Biology coordinated a symposium titled “Sexual Diversity and Variation” (2023) specifically aiming to address if sexual diversity could be accurately captured by a male-female binary. The resulting publications [(Lewis & Sharpe 2023; McLaughlin *et al.* 2023; Sharpe *et al.* 2023)](https://paperpile.com/c/d0sRtm/0EhSj+tTxED+SggyP) proposed frameworks for multivariate approaches to sex. Similarly, Cell’s special issue “Focus on Sex and Gender” (2024) featured multiple papers debating the utility of defining sex in science [(Aghi *et al.* 2024; Joyce *et al.* 2024; Pape *et al.* 2024; Velocci 2024)](https://paperpile.com/c/d0sRtm/nJE8j+i1JaV+77g6H+nZRny) along with a rollout of guidelines for reporting sex- and gender-based analyses in *Cell* Journals [(Cell Press 2024)](https://paperpile.com/c/d0sRtm/C8lWx). These guidelines aim to “enhance reporting precision, encouraging authors to consider the nuances of sex and gender and choose the most accurate terms so readers can better understand what they are assessing” because “with sex and gender, there are so many definitions" [(Cell Press 2023)](https://paperpile.com/c/d0sRtm/8GWsh). Further, the Society for the Study of Evolution (SSE), American Society of Naturalists (ASN), and Society of Systematic Biologists (SSB) (Tri-Societies) hosted a “Teaching Sex and Gender” Symposium at the in-person 2025 meeting to “explore the current science behind sex and gender, explore how educators can move their instruction beyond simple binary XX/XY paradigms, and provide educational materials for teaching this nuanced and difficult subject”. These formal debates are taking place in mainstream venues and prove continual engagement with definitional questions surrounding biological sex.

**Professional Society Debates:** In response to U.S. executive order 14168, which aims to categorize sex as a binary and immutable fact of biology, the Tri-societies published a public letter to the President and the Congress of the United States in February 2025 that stated "sex… is not a binary trait" and "scientific consensus defines sex … as a biological construct that relies on a combination of chromosomes, hormonal balances, and the resulting expression of gonads, external genitalia and secondary sex characteristics" [(Boggs et al. 2025)](https://paperpile.com/c/GyFKgw/nMHR). This letter, written by the three presidents of the tri-societies (which represent over 3,500 scientists), was sent to the President of the United States and Members of the U.S. Congress, then subsequently retracted due to internal debates about the definition of sex among society members. This letter is not the first from the tri-societies, indeed a similar letter signed by >2,600 scientists, ASN, SSE, and SSB (again representing over 3,500 scientists) was published in 2018 to Secretary Azar on the Scientific Understanding of Sex and Gender, which stated “Variation in biological sex and in gendered expression has been well documented in many species, including humans, through hundreds of scientific articles. Such variation is observed at both the genetic level and at the individual level (including hormone levels, secondary sexual characteristics, as well as genital morphology). Moreover, models predict that variation should exist within the categories that HHS [Department of Human and Health Services] proposes as "male" and “female”, indicating that sex should be more accurately viewed as a continuum” [(Hoekstra et al. 2018;](https://paperpile.com/c/GyFKgw/esOC+tqG4) [Not-binary.org](http://not-binary.org) [2018)](https://paperpile.com/c/GyFKgw/esOC+tqG4).

A counter-signature campaign by dissenting members, including a past SSE president, explicitly rejected this characterization and advocated for the gametic definition as the sole definition of biological sex [(Coyne & Maroja 2025; Maroja 2025)](https://paperpile.com/c/d0sRtm/5XPna+DchJs). Within their letter of dissent, this group of >130 scientists stated: “The point of *this* letter is not to show that our view is a ‘consensus’ (the Societies did not poll their members, either) but simply to affirm that a variety of people in biology or adjacent areas reject the Societies’ construal of sex as both a ‘construct’ and a ‘spectrum’”. Further pointing out the lack of consensus in biological sex definitions, the letter stated “The Tri-societies were wrong to speak in our names and claim that there is a scientific consensus without even conducting a survey of society members to see if such a consensus exists”. This debate is also not isolated to the tri-societies nor the field of ecology and evolutionary biology; a similar letter was published by the American Anthropological Association in 2023, using the multi-variate definition of sex, which ultimately led to a withdrawn session about the topic of biological sex at their annual meeting [(Fuentes et al. 2023)](https://paperpile.com/c/GyFKgw/vsOc).

**Philosophical and Biological Literature:** The definition and conceptual boundaries of biological sex have been debated for decades in the philosophical and biological literature by a number of high-profile works, including but not limited to [(](https://paperpile.com/c/GyFKgw/C6QYD+fz82+7Ewg)[Ainsworth 2015](https://paperpile.com/c/GyFKgw/Hpiv); [Griffiths 2020; Griffiths and Spencer 2025; Roughgarden 2004)](https://paperpile.com/c/GyFKgw/C6QYD+fz82+7Ewg). In addition, every paper published in ecology and evolutionary biology with an empirical study system that defines sex operationally (*i.e.,* by a defining trait that is directly observed within the system), actively contributes to the discourse. In recent years, this debate has been explicitly identified, for instance in [(McLaughlin et al. 2023)](https://paperpile.com/c/GyFKgw/7sYd), which states “Whereas some biologists argue that gametes are the only meaningful sex categories [(Goymann et al. 2023)](https://paperpile.com/c/GyFKgw/18ee), we find several limitations to the gametic sex definition, particularly for ecologists and evolutionary biologists.”

**References**

Aghi, K., Anderson, B.M., Castellano, B.M., Cunningham, A., Delano, M., Dickinson, E.S., *et al.* (2024). Rigorous science demands support of transgender scientists. *Cell*, 187, 1327–1334.

Ainsworth, C. (2015). Sex redefined. *Nature*, 518, 288–291.

Boggs, C., Bolnick, D. & Ware, J. (2025). Policy: Letter to the US president and congress on the scientific understanding of sex and gender.

Cell Press. (2023). *Author guideline updates address how to consider language related to “sex” and “gender.”* Available at: https://www.cell.com/news-do/sex-and-gender-guidelines.

Cell Press. (2024). *Landmark Cell focus issue centers the voices of sex and gender minorities*. Available at: https://www.cell.com/news-do/cell-sex-gender-focus-issue.

Coyne, J. & Maroja, L.S. (2025). A group letter to the presidents of three evolution/ecology societies objecting to their characterization of sex as a spectrum in humans and all other species.

Goymann, W., Brumm, H. & Kappeler, P.M. (2023). Biological sex is binary, even though there is a rainbow of sex roles: Denying biological sex is anthropocentric and promotes species chauvinism: Denying biological sex is anthropocentric and promotes species chauvinism: Denying biological sex is anthropocentric and promotes species chauvinism. *Bioessays*, 45, e2200173.

Griffiths, P. (2020). *The existence of biological sex is no constraint on human diversity*. *Aeon*. https://aeon.co/essays/the-existence-of-biological-sex-is-no-constraint-on-human-diversity.

Griffiths, P.E. & Spencer, H.G. (2025). Biology should not dispense with sexes. *Curr. Biol.*, 35, R244–R248.

Hoekstra, H., Strauss, S. & Magallón, S. (2018). Letter to Secretary Azar on the Scientific Understanding of Sex and Gender. https://www.amnat.org/announcements/LTRgender.html.

Joyce, J.A., Masina, S., Michalik, L., Pot, C., Sempoux, C. & Amati, F. (2024). Closing the scissor-shaped curve: Strategies to promote gender equality in academia. *Cell*, 187, 1335–1342.

Lewis, A.K. & Sharpe, S.L. (2023). Sex, Science, and Society: Reckonings and Responsibilities for Biologists. *Integr. Comp. Biol.*, 63, 877–885.

Maroja, L. (2025). *Sex in Biology Signature Campaign*. *Heterodox STEM*. Available at: https://hxstem.substack.com/p/sex-in-biology-signature-campaign.

Fuentes, A., Clancy, K., & Nelson, R. (2023). “Letter of Support for AAA’s Withdrawal of Session from the Annual Meeting.” The American Anthropological Association. https://americananthro.org/news/letter-of-support-for-aaas-withdrawal-of-session-from-the-annual-meeting/.

McLaughlin, J.F., Brock, K.M., Gates, I., Pethkar, A., Piattoni, M., Rossi, A., *et al.* (2023). Multivariate models of animal sex: Breaking binaries leads to a better understanding of ecology and evolution. *Integr. Comp. Biol.*, 63, 891–906.

Not-Binary.org. (2018). “Transgender, Intersex, and Gender Non-Conforming People #WontBeErased by Pseudoscience.” https://not-binary.org/statement/.

Pape, M., Miyagi, M., Ritz, S.A., Boulicault, M., Richardson, S.S. & Maney, D.L. (2024). Sex contextualism in laboratory research: Enhancing rigor and precision in the study of sex-related variables. *Cell*, 187, 1316–1326.

Roughgarden, J. (2004). *Evolution’s Rainbow Diversity, Gender, and Sexuality in Nature and People*. 1st edn. University of California Press.

Sharpe, S.L., Anderson, A.P., Cooper, I., James, T.Y., Kralick, A.E., Lindahl, H., *et al.* (2023). Sex and biology: Broader impacts beyond the binary. *Integr. Comp. Biol.*, 63, 960–967.

Velocci, B. (2024). The history of sex research: Is “sex” a useful category? *Cell*, 187, 1343–1346.
